# Rubidium-82 PET imaging is feasible in a rat myocardial infarction model

**DOI:** 10.1007/s12350-017-0994-9

**Published:** 2017-07-18

**Authors:** Adam Ali Ghotbi, Andreas Clemmensen, Kasper Kyhl, Bjarke Follin, Philip Hasbak, Thomas Engstrøm, Rasmus Sejersten Ripa, Andreas Kjaer

**Affiliations:** 1 Department of Clinical Physiology, Nuclear Medicine & PET and Cluster for Molecular Imaging, Rigshospitalet and University of Copenhagen, Blegdamsvej 9, 2100 Copenhagen, Denmark; 2 Department of Cardiology, The Heart Center, Rigshospitalet and University of Copenhagen, Copenhagen, Denmark; 3 Cardiology Stem Cell Center, The Heart Center, Rigshospitalet and University of Copenhagen, Blegdamsvej 9, 2100 Copenhagen, Denmark

**Keywords:** Small-animal heart, rat myocardium, perfusion imaging, infarction, rubidium-82 PET, magnetic resonance

## Abstract

**Background:**

Small-animal myocardial infarct models are frequently used in the assessment of new cardioprotective strategies. A validated quantification of perfusion using a non-cyclotron-dependent PET tracer would be of importance in monitoring response to therapy. We tested whether myocardial PET perfusion imaging is feasible with Rubidium-82 (^82^Rb) in a small-animal scanner using a rat myocardial infarct model.

**Methods:**

18 Sprague-Dawley rats underwent permanent coronary artery ligation (infarct group), and 11 rats underwent ischemia-reperfusion (reperfusion group) procedure. ^82^Rb-PET and magnetic resonance imaging (MRI) were conducted before and after the intervention. Perfusion was compared to both left ventricle ejection fraction (LVEF) and infarct size assessed by MRI.

**Results:**

Follow-up global ^82^Rb-uptake correlated significantly with infarct size (infarct group: *r* = −0.81, *P* < 0.001 and reperfusion group: *r* = −0.61, *P* = 0.04). Only ^82^Rb-uptake in the infarct group correlated with LVEF. At follow-up, a higher segmental ^82^Rb-uptake in the infarct group was associated with better wall motion (*β* = 0.034, CI [0.028;0.039], *P* < 0.001, *R*^2^ = 0.30), and inversely associated with scar transmurality (*β* = −2.4 [−2.6; −2.2], *P* < 0.001, *R*^2^ = 0.59). The associations were similar for the reperfusion group.

**Conclusion:**

^82^Rb-PET is feasible in small animal scanners despite the long positron range and enables fast and time-efficient myocardial perfusion imaging in rat models.

**Electronic supplementary material:**

The online version of this article (doi:10.1007/s12350-017-0994-9) contains supplementary material, which is available to authorized users.

## Introduction

Small-animal heart models, e.g., rat or mouse, are widely used to explore pathophysiology and test new cardioprotective modalities in the infarct and ischemia-reperfusion settings.^1^,^2^ To this end, it is necessary to establish the infarct area. Histological techniques are available, but their application is limited since multiple, and longitudinal assessments of the myocardial injury are not possible.^3^ Non-invasive techniques are preferred because they allow serial assessment in vivo. Magnetic resonance imaging (MRI) provides precise anatomic and functional assessments and is considered the gold standard in infarct assessment.^4^ However, the modality is time-consuming and limited in assessing physiologic aspects of the disease phenotype.^5^,^6^ Single-photon emission tomography (SPECT) with ^99m^Tc-sestamibi imaging is also available, however, hampered by low time efficiency.^7^,^8^

Feasibility of myocardial perfusion imaging with positron emission tomography (PET) has previously been demonstrated in murine models with the ^13^N-ammonia tracer. However, this tracer requires cyclotron and is rather time-consuming to produce, which curbs its usage.^9^–^11^ The usage of Rubidium-82 (^82^Rb) in the clinical setting is well established and could prove to be an alternative perfusion tracer in small animals despite its long positron range compared to other frequently used cardiac PET tracers.^12^ The high cost of the ^82^Rb-generator potentially limits its use in preclinical research. However, a generator can only be used clinically up to a maximum of 42 days after calibration. At this time point, these clinically expired generators still elute enough ^82^Rb to perform preclinical MPI-PET for another 5 to 6 weeks. This setup can keep the price of the tracer for preclinical research at a reasonable level. The short half-life of ^82^Rb permits fast sequential perfusion imaging and high imaging throughput^12^ compared to other imaging techniques such as MRI.

Therefore, the aim of our study was to explore the feasibility of ^82^Rb-imaging in rats undergoing left anterior descending artery (LAD) ligation in a dedicated small-animal PET scanner. We hypothesized that rat myocardial perfusion uptake of ^82^Rb correlate with MRI-derived left ventricular ejection fraction (LVEF) and infarct size and predicts segmental wall motion and late gadolinium enhancement (LGE).

## Methods

**Figure 1:**
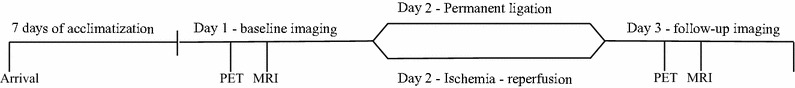
Study design. *Arrival*, of animals to the core laboratory; *PET*, positron emission tomography; *MRI*, magnetic resonance imaging

All animal experiments were approved by the Danish Animal Experiments Inspectorate (Permit No. 2012-15-2934-00064). The animals were cared for at core animal facilities at 21 ± 2 °C with a 12:12 hours dark:light cycle, were weighed once daily, and had access to water and rodent food ad libitum. The animals were acclimatized for 5 to 12 days before being included. For study design see Figure 1.

### Study Outline

A total of 29 male Sprague-Dawley rats, 6 to 7 weeks old, weighing 256 grams [interquartile range (IQR): 243; 263] were divided into two groups: 18 with permanent proximal LAD ligation and 11 with ischemia duration for 35 minutes followed by reperfusion. The study design is shown in Figure 1. Myocardial infarction (MI) was induced as described elsewhere.^13^ In brief, anesthesia was commenced with 4% sevoflurane and continued at 2.5% to 3%. Rats were intubated and ventilated (UNO micro-ventilator-03, Netherlands) and were placed on a heated surface with ECG monitoring. The rats were pain treated with Buprenorphine 0.05 mg/kg at the intervention and three times daily the next 3 days. Thoracotomy was performed under surgical standards at the fourth intercostal space. The pericardium was opened, and the LAD ligated caudal of its origin with a 6-0 polypropylene suture. The ischemia-reperfusion rats underwent a transient LAD ligation, where the suture was looped and tighten around a polyethylene tubing (diameter 2.7 mm). The ligature was released and the tubing removed after the ischemia period (35 minutes). Ischemia was confirmed by discoloration of the myocardium and ECG changes. Reperfusion was visually confirmed. The chest, muscle layers, and skin were closed with vicryl 4.0 suture. Sham operation was performed on two rats as described above except no LAD ligation took place.

### PET Imaging

All animals underwent a baseline scan one-day prior and a follow-up scan one-day after the intervention, respectively. Imaging was performed on a dedicated preclinical PET/computed tomography (CT) scanner (Siemens Inveon, Knoxville, TN, USA). Rats were anesthetized with sevoflurane 4% in a chamber and maintained with inhaled 2% to 3.5% sevoflurane mixed with air/oxygen; the dorsal tail vein was cannulated with a permanent catheter. The rats were placed in prone position on water heated imaging bed with ECG, respiration and temperature monitoring. An initial scout CT imaging insured correct positioning. A newly expired clinical ^82^Rb-generator (CardioGen-82, Bracco Diagnostics Inc., Monroe Township, NJ, USA) was calibrated according to vendor requirements and equipped with a 3-way valve to control amount of infusion to the rats. Approximately 100 mega Becquerel (MBq) of ^82^Rb in 1.25 to 1.5 mL saline solution was administered with simultaneous list mode PET acquisition for 5 minutes. For attenuation correction and anatomical co-registration, a CT scan was acquired subsequently. In addition, two rats underwent CT with contrast (Omipaque 350 mg/mL, 1.5 mL intravenously) to validate the use of the non-contrast-enhanced CT in the remaining rats for correct anatomical alignment of the ^82^Rb-PET examination. PET list-mode data were histogrammed into two static timeframes, the first 45 seconds and the remaining 255 seconds. The latter timeframe was used for the analyses presented in this paper. This was done to eliminate ^82^Rb-activity from the blood pool and minimize the spillover effect. The images were reconstructed non-gated using the vendor supplied ordered Poison—maximum a posteriori probability (OP-MAP)/3D-ordered subset expectation maximization (OSEM) algorithm with 3 mm requested resolution, 2 iterations, and 18 subsets, respectively. Images were scatter, attenuation and prompt-gamma corrected.^13^

### PET Image Analysis

PET data were processed and analyzed in PMOD cardiac tool version 3.3 (PMOD technologies LLC, Zurich, Switzerland). The images were first re-orientated into short, vertical, and horizontal long-axis. Next, the myocardial contour was determined in a semi-automatic manner by applying marker guidance on basal and apical points of the left ventricle (LV). The markers were placed according to extrapolation, and by fusion of PET with CT examination with and without i.v. contrast where the basal and apical points were visualized. The contours produced by the software were then assessed visually by an experienced operator (AG) and corrected manually if needed. ^82^Rb-uptakes were indexed to maximum uptake. Subsequently, polar maps of the LV were created and segmented into basal, mid and apical sections similar to the American Heart Association (AHA) 17-segment model.^14^ To examine the intra-observer variability, were all rat data reexamined on two separate days. All data analysis was performed blinded to treatment.

### MRI Imaging

MRI was performed within an hour from PET imaging using a 7 Tesla preclinical scanner (Bruker Pharmascan, Bruker Medical, Ettlingen, Germany) with a horizontal bore and a 60-mm transmitter–receiver coil. The rats were transferred from the PET scanner (or vice versa) in anesthesia onto a bed with similar design and monitoring as described with the PET imaging. Scout imaging (3 slices, 1 mm thickness, field of view 3.5 × 3.5 mm^2^, repetition time (TR) 85 ms, echo time (TE) 1.5 ms) was utilized to verify correct positioning, and to produce 2- and 4-chamber long axis images. Short axis images were acquired perpendicular to the 2- and 4-chamber images. Next, a stack of retrospectively self-gated (Intragate) FLASH (fast low-angle shot) cine-sequence in short axis view (perpendicular to the 2- and 4-chamber images) was used to cover the entire heart in 1-mm-thick slices with 0.5-mm gap in between. Field of view was 4.5 × .4.5 cm^2^ with a 256 × 256 matrix size, TR 4.8 ms, TE 2.0 ms, bandwidth 4566.7 Hz, 150 number of repetitions, flip-angle 10°. We obtained 15 phases during the cardiac cycle. Fifteen minutes after administration of gadolinium contrast (diethylenetriamine pentacetic acid, 0.1 mol/kg, Gadovist, Bayer Scherring Pharma), a multislice ECG-gated FLASH sequence was acquired to delineate the enhanced areas in the LV at end-diastolic phase of the ECG with respiratory gating. The sequence parameters were as follows: slice thickness 1 mm, slice gap 0.5 mm, field of view 6 × 6 cm^2^, matrix size 256 × 256, TR 70 ms, TE 2.8 ms, flip-angle 30°.^15^

### MRI Image Analysis

The short-axis cine images were used to calculate LVEF, end-diastolic (EDV), and end-systolic volumes (ESV), as well as wall-motion and wall-thickening on AHA-17 segments. To obtain this, the largest (EDV) and smallest LV (ESV) volume were determined manually on the 15 framed cardiac cycle; the endo- and epicardial contours of these frames were manually traced using dedicated software (CVI42 v. 4.0.1, Circle Cardiovascular Imaging, Calgary, Canada). Polar maps were generated automatically from the software.

The endo- and epicardial contours of the LGE images were manually traced. Infarct size was assessed on follow-up LGE images and defined as hyperintensive myocardium 5 standard deviations above the mean value in a region-of-interest in normal myocardium. Isolated hypointensive areas within the enhanced region were considered as part of the infarct size. Infarct size was expressed as percent of the LV volume. The operator (AG) was blinded to PET data. To examine the inter-observer variability, a second operator (KK) analyzed 10 randomly selected rat datasets (blinded to the results of AG).

### Statistics

Continuous variables are presented as median [interquartile range, IQR] and categorical data as frequencies or percent (%). For comparison, the non-parametric Wilcoxon matched-pairs signed rank was used. Spearman’s correlation was used when comparing global outcomes between the two modalities. Linear mixed models were used for the comparison on segmental level in order to account for correlation between segments belonging to the same animal. *R*^2^ was calculated as described by Nakagawa et al^16^ A two-sided *P* value <0.05 was considered significant. All statistical analyses were performed using SPSS^®^ version 19 (IBM SPSS, Chicago, IL, USA).

## Results

The typical PET/CT acquisition time was 10 to 15 minutes. Baseline and follow-up PET and MRI imaging were performed on average 23 hours before and after the intervention, respectively. See Table 1.

**Table 1:** Baseline characteristics

	RAT
N	29 PL 18 IR 11
Age, weeks [IQR]	6.8 [6.4; 7.1]
Weight, grams	256 [243; 263]
Tibia length, mm	46.9 [45.4; 47.5]
Baseline, time from PET to MRI, hh:mm	0:57 [0:40; 1:40]
Baseline PET to ligation, hh:mm	23:30 [22:23; 25:05]
Follow-up, ligation to PET, hh:mm	23:50 [22:30; 25:05]
Follow-up, PET to MRI, hh:mm	0:50 [0:30; 1:15]
Ischemia duration hh:mm*	0:35 [0:35; 0:35]
^82^Rb injected, MBq	
Baseline	100 [72; 126]
Follow-up	103 [88; 122]

Values are median [IQR] interquartile range

*PET*, positron emission tomography; *MRI*, magnetic resonance imaging; *PL*, permanent ligation; *IR*, ischemia–reperfusion;^*82*^*Rb*, Rubidium-82; *MBq*, mega Becquerel

* Only for ischemia–reperfusion intervention rats

### Image Quality

**Figure 2:**
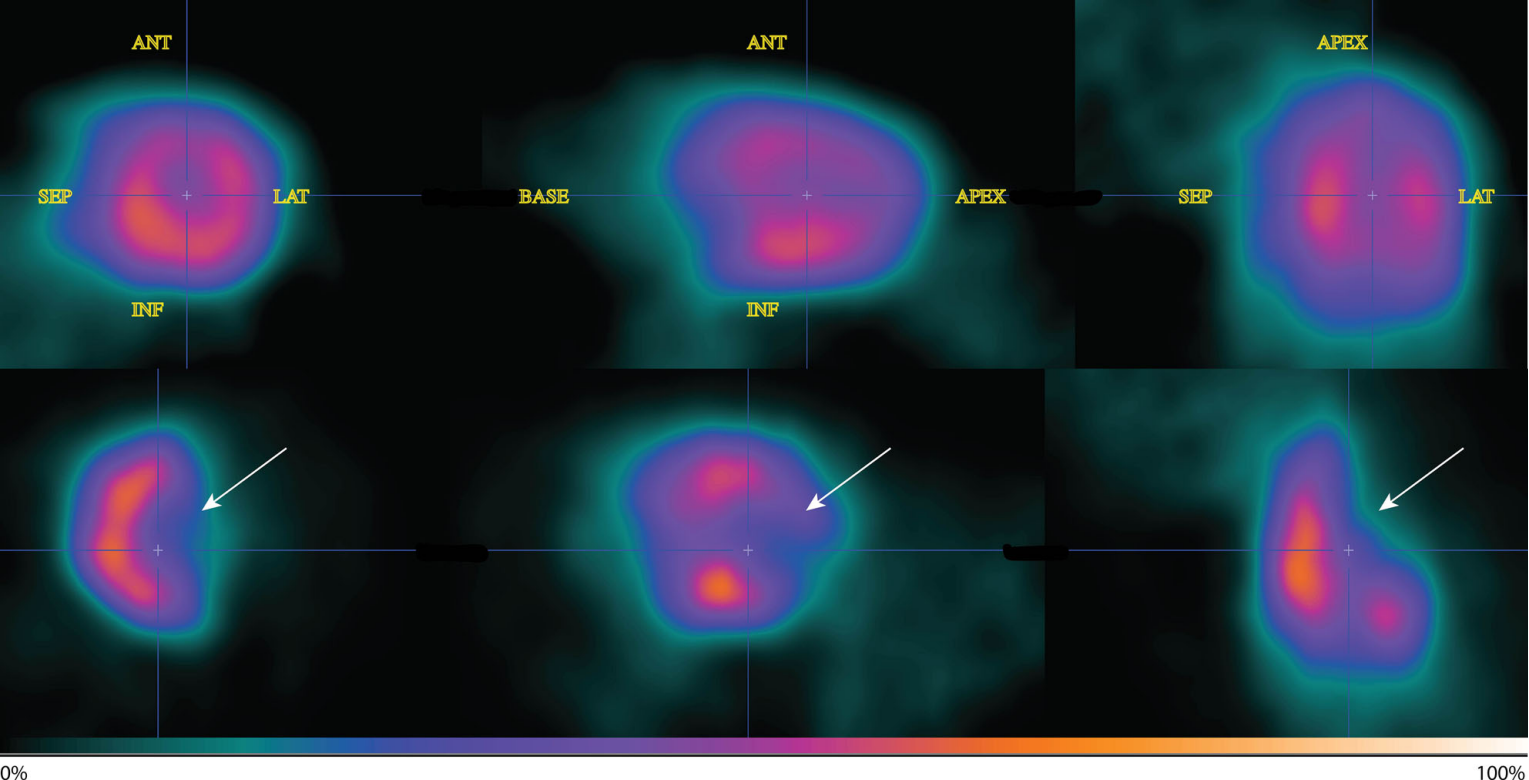
*Upper panel* baseline ^82^Rb-imaging, *lower panel* follow-up imaging after intervention. From *left* to *right*: short axis, *horizontal* and *vertical view* of the *left* ventricle of rat with permanent *left* anterior descending artery ligation. *White arrows* point to the decreased ^82^Rb-uptake at the anterolateral wall. *ANT*, anterior; *LAT*, lateral; *SEP*, septum; *INF*, inferior. *Color* scale indicates tracer uptake (i.e., 0% to 100%)

Representative images of pre- and post-intervention of ^82^Rb-uptake are shown in Figure 2. The images were of average to good diagnostic quality, i.e., ability to identify the LV walls and MI. However, a pattern of better image quality was observed after the intervention, probably due to dilatation of the LV. Average total true counts per image were 60 × 10^6^ [50 × 10^6^; 71 × 10^6^] (Table 2).

**Table 2:** MRI parameters before and after intervention

	MRI		
	Baseline imaging	Follow-up imaging	*P* value
LVEF, % [IQR]			
PL	61.0 [58.9; 63.6]	30.0 [23.0; 39.1]	<0.001
IR	60.5 [58.5; 64.2]	43.5 [36.8; 57.8]	=0.002
EDV, mL.			
PL	0.449 [0.398; 0.505]	0.519 [0.470; 0.587]	=0.02
IR	0.460 [0.419; 0.496]	0.442 [0.379; 0.526]	ns
ESV, mL.			
PL	0.175 [0.142; 0.198]	0.395 [0.314; 0.426]	<0.001
IR	0.177 [0.156; 0.198]	0.249 [0.202; 0.307]	=0.004
Infarct size, % of LV			
PL	na.	34.7 [19.7; 44.5]	
IR		21.7 [12.0; 29.6]	

Values are median [IQR] interquartile range

*MRI*, magnetic resonance imaging; *LVEF*, left ventricle ejection fraction; *EDV*, end-diastolic volume; *ESV*, end-systolic volume; *PL*, permanent ligation; *IR*, ischemia–reperfusion; *na*, not available; *ns*, non-significant

### PET and MRI Results

Rats in the permanent ligation group had a modest, but significant decrease in total LV myocardial ^82^Rb-uptake at follow-up compared to baseline. The apical segment showed a significant reduction in ^82^Rb-uptake for both the permanent ligation (80.1% [73.4; 90.3] vs. 89.9% [87.2; 91.7], *P* < 0.005) and the ischemia-reperfusion (84.9% [79.5; 88.5] vs. 88.2% [87.3; 89.4], *P* = 0.02) groups at follow-up compared to baseline imaging. See Table 3.

**Table 3:** Indexed ^82^Rb uptake in rat apical, mid, and basal segments of the heart before and after intervention

^82^Rb-uptake	Baseline imaging	Follow-up imaging	*P* value
Total			
PL	93.6 [92.0; 94.9]	87.6 [83.1; 94.3]	=0.01
IR	92.8 [92.3; 93.6]	91.7 [89.3; 94.0]	=ns
Apical			
PL	89.9 [87.2; 91.7]	80.1 [73.4; 90.3]	<0.005
IR	88.2 [87.3; 89.4]	84.9 [79.5; 88.5]	=0.02
Mid			
PL	93.6 [92.7; 95.5]	87.9 [82.1; 94.3]	=0.007
IR	93.1 [91.5; 93.9]	91.2 [89.6; 93.8]	=ns
Basal			
PL	96.2 [95.6; 97.6]	93.5 [91.5; 96.6]	=0.02
IR	96.2 [95.9; 97.0]	96.8 [95.6; 97.7]	=ns

Values are median [IQR] interquartile range

^*82*^*Rb*, rubidium-82;* PL*, permanent ligation; *IR*, ischemia–reperfusion; *ns*, non-significant

There was no difference in LVEF or LV dimensions between the permanent ligation and the ischemia-reperfusion group prior to the intervention. After the intervention, the LVEF of the permanent ligation group was reduced to 30% [23; 39], while the ischemia-reperfusion group was reduced to 44% [37; 58], compared to 61% [59; 64] at baseline, *P* < 0.001 and *P* = 0.002, respectively. Infarct size was estimated to 34.7% [19.7; 44.5] of the LV in the permanent ligation group, while it amounted to 21.7% [12.0; 29.6] of the LV in the ischemia-reperfusion group. See Table 2.

The sham rats did not exhibit any changes at post-intervention PET and MRI imaging.

### PET and MRI Comparison

**Figure 3:**
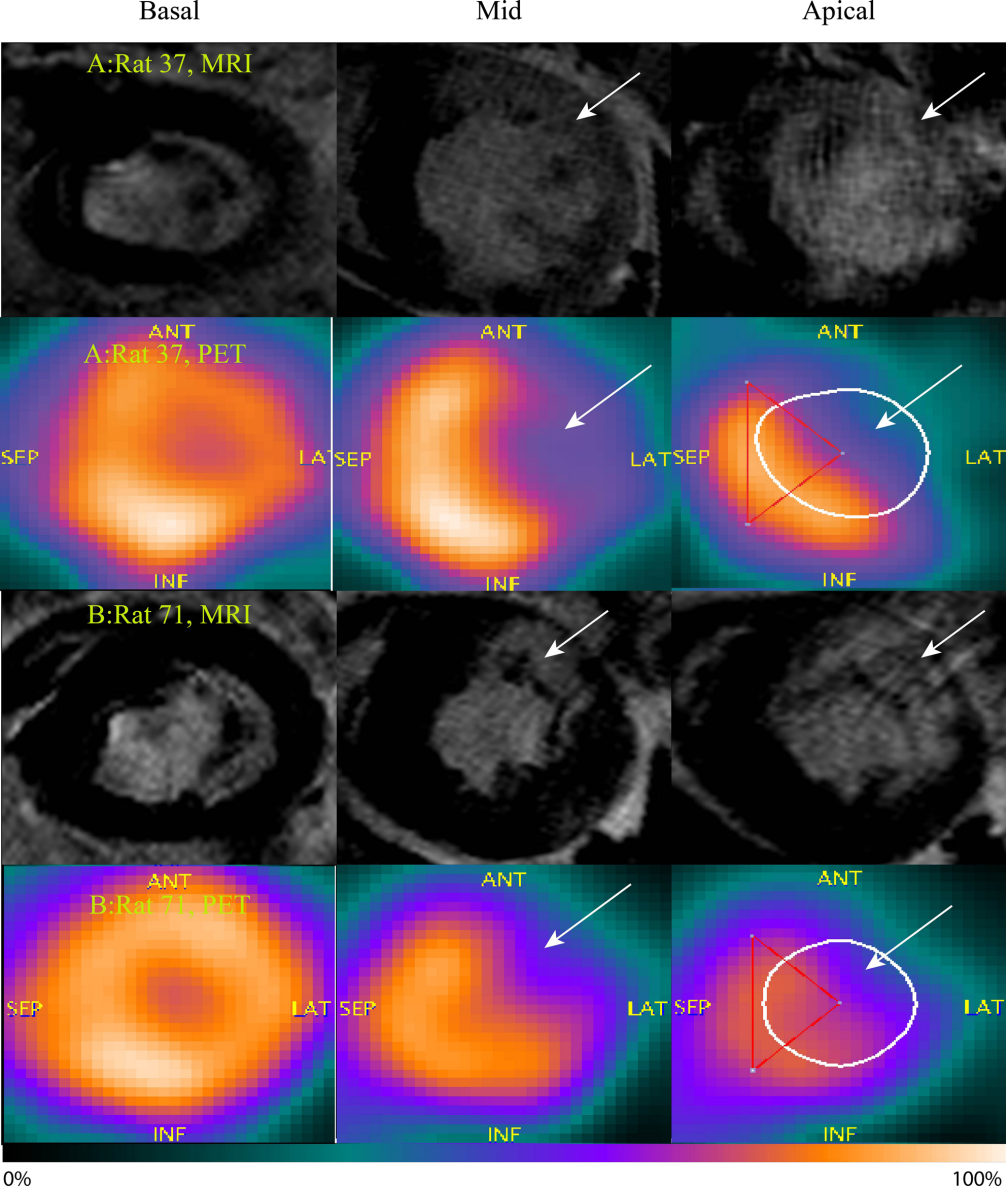
Comparison between MRI and ^82^Rb-PET. *Two upper panels***A** represent basal, mid and apical short-axis view of the *left* ventricle of a rat with permanent ligation with MRI and PET. Two *lower panels***B** represent a rat with ischemia-reperfusion. *White arrows* point to areas with late gadolinium enhancement on the MRI and decreased ^82^Rb-uptake on PET images. *ANT*, anterior; *LAT*, lateral; *SEP*, septum; *INF*, inferior *Color* scale indicates tracer uptake (i.e., 0% to 100%)

**Figure 4:**
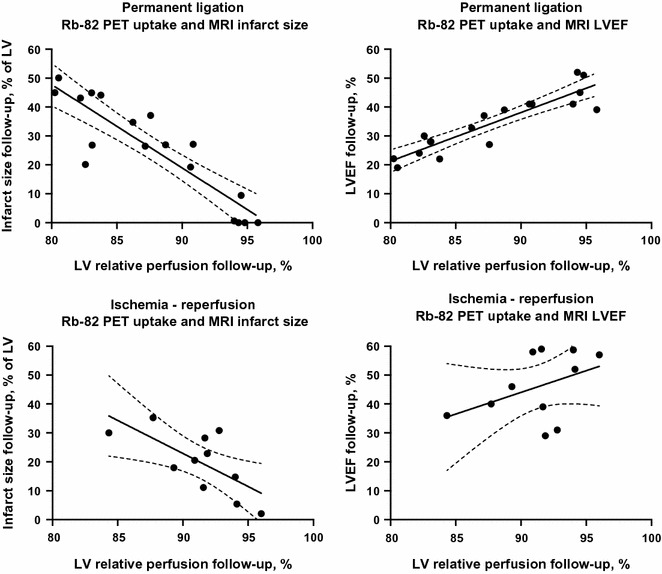
*Upper panel* permanent ligated rats. *Lower panel* ischemia-reperfusion rats. Global ^82^Rb-uptake and MRI-derived infarct size, and global ^82^Rb-uptake and MRI-derived *left* ventricle ejection fraction. ^*82*^*Rb*, rubidium-82; *MRI*, magnetic resonance imaging; *LVEF*, left ventricle ejection fraction

The total LV and apical segment follow-up ^82^Rb-myocardial uptake in the permanent ligation group correlated strongly with follow-up MRI-derived LVEF (*r* = 0.89, *P* < 0.001 for both parameters) and with infarct size (*r* = −0.81, *P* < 0.001 and *r* = −0.78, *P* < 0.001, respectively). See Figures 3, 4 and Table 4. Meanwhile, in the ischemia–reperfusion group, the follow-up total LV(*r* = −0.61, *P* = 0.04) and apical (*r* = −0.66, *P* = 0.02) ^82^Rb-uptake correlated moderately with follow-up infarct size. In contrast, the total LV ^82^Rb-uptake did not correlate significantly with follow-up LVEF.

**Table 4:** Correlation between ^82^Rb uptake and post-intervention MRI

	MRI follow-up	
^82^Rb-uptake follow-up	LVEF, %	Infarct size, %
Total		
PL	*r* = 0.89, *P* < 0.001	*r* = −0.81, *P* < 0.001
IR	*r* = 0.19, *P* = ns	*r* = −0.61, *P* = 0.04
Apical		
PL	*r* = 0.89, *P* < 0.001	*r* = −0.78, *P* < 0.001
IR	*r* = 0.40, *P* = ns	*r* = −0.66, *P* = 0.02
Mid		
PL	*r* = 0.91, *P* < 0.001	*r* = −0.85, *P* < 0.001
IR	*r* = 0.28, *P* = ns	*r* = −0.60, *P* = ns
Basal		
PL	*r* = 0.44, *P* = ns	*r* = −0.34, *P* = ns
IR	*r* = −0.16, *P* = ns	*r* = −0.36, *P* = ns

*LVEF*, left ventricle ejection fraction; *PL*, permanent ligation; *IR*, ischemia–reperfusion; *ns*, non-significant

### Segmental PET and MRI Comparison

**Figure 5:**
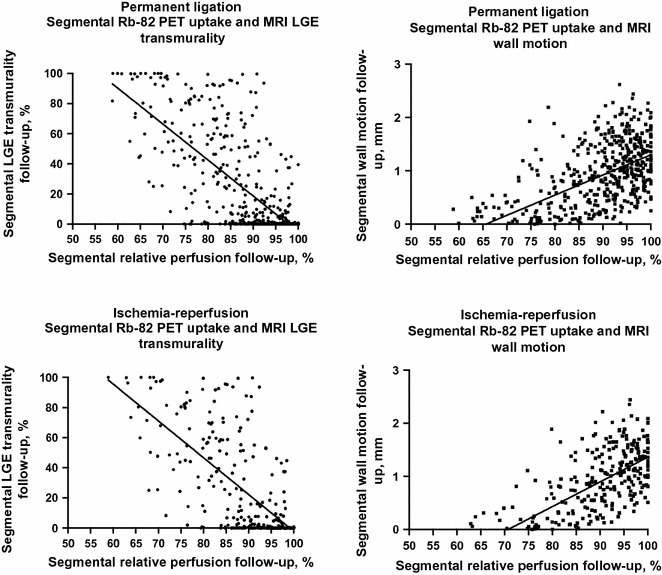
*Upper panel* permanent ligated rats. *Lower panel* ischemia-reperfusion rats. Segmental ^82^Rb-uptake and MRI late gadolinium enhancement, and segmental ^82^Rb-uptake and MRI wall motion. ^*82*^*Rb*, rubidium-82; *MRI*, magnetic resonance imaging; *LGE*, late gadolinium enhancement; *mm*, millimeters

Comparing follow-up ^82^Rb-uptake according to the AHA-17 segments with follow-up MRI derivatives of wall motion and LGE transmurality per AHA-17 segments yielded also strong associations: in the permanent ligation group, a higher ^82^Rb-uptake was associated with higher wall motion (*β* = 0.034, CI [0.028;0.039], *P* < 0.001, *R*^2^ = 0.30), and inversely associated with lower degree of LGE transmurality (*β* = −2.4 [−2.6; −2.2], *P* < 0.001, *R*^2^ = 0.59). The associations were similar when comparing the ischemia–reperfusion group ^82^Rb-uptake with the same MRI parameters, see Table 5 and Figure 5.

**Table 5:** Linear Mixed Models

Outcome	Rat MRI follow-up no explanatory factors except PET derivatives								
	Wall thickening (% AHA-17 segments)			Wall Motion (mm AHA-17 segments)			LGE (% transmurality per AHA-17 segments)		
PET	Beta	*P*	*R* ^2^	Beta	*P*	*R* ^2^	Beta	*P*	*R* ^2^
Total LV ^82^Rb-uptake PL group (%)	0.32 [0.02; 0.61]	0.04*	0.01	0.034 [0.028; 0.039]	0.001*	0.30	−2.4 [−2.6; −2.2]	0.001*	0.59
Total LV ^82^Rb-uptake IR group (%)	0.4 [−0.08; 0.89]	ns	0.01	0.05 [0.04; 0.06]	0.001*	0.34	−2.7 [−3.2; −2.2]	0.001*	0.35

*LVEF*, left ventricle ejection fraction; *PL*, permanent ligation; *IR*, ischemia–reperfusion; *ns*, non-significant; *AHA*, American Heart Association; *LGE*, late gadolinium enhancement

### Reproducibility

The intra-observer interclass correlation coefficient was above 0.8 for all parameters. A subset of 10 randomly selected rat MRI data was read by two readers (AG & KK). The inter-observer interclass correlation coefficients were between 0.76 and 0.98, see Table 6.

**Table 6:** Inter-observer and intra-observer correlation

MRI data	LVEF baseline	LVEF follow-up	Infarct size
Inter-observer ICC (10 datapoints)	0.810 [0.237;0.953], *P* = 0.01	0.981 [0.904;0.996], *P* < 0.001	0.767 [0.163;0.953], *P* = 0.04

PET data	Total LV ^82^Rb-uptake baseline	Total LV ^82^Rb-uptake follow-up	Basal ^82^Rb-uptake post
Intra-observer ICC (29 datapoints)	0.899 [0.786;0.953], *P* < 0.001	0.899 [0.788;0.952], *P* < 0.001	0.828 [0.639; 0.918], *P* < 0.001

Inter-observer and intra-observer (ICC) = interclass correlation for MRI and PET data

## Discussion

In this study, we investigated the feasibility of ^82^Rb-PET in small-animal heart models of permanent and transient ischemia and compared the PET results with MRI derivatives. The results suggest that despite the high positron range, ^82^Rb-perfusion imaging of the post-ischemic phase is feasible in rats and that it is correlated with LVEF, infarct size, wall motion and LGE transmurality derived from MRI.

Developing and testing novel cardioprotective treatment requires translation from preclinical validation. The small-animal heart model offers a relatively inexpensive and logistical manageable setup, where markers such as infarct size, myocardial perfusion defect, and LVEF can measure the efficacy of new treatment regiments. Thus, reliable, repeatable and time-efficient non-invasive measurements of the myocardium in rodents are valuable.^11^ Considering our results and the high degree of intra- and inter-observer reproducibility, the ^82^Rb-imaging could prove to be a valuable tool in evaluating myocardial perfusion in rats and allow assessment of large number of scans in a time-efficient manner. However, further studies are needed to establish the repeatability of ^82^Rb-perfusion imaging in small animals.

The combination of rapid heart rate, respiration, and thin myocardial wall combined with the long positron range of the ^82^Rb makes assessment of the rat heart challenging due to intrinsic blurring and activity spillover from the blood pool.^10^ Furthermore, absolute blood flow quantification requires time-activity curves for the blood pool and the myocardium, which is difficult due to the contamination of activity across the compartments. The visual quality of the ^82^Rb images was moderate, but nonetheless, allowed clear delineation of the myocardial wall and perfusion defects, which were strongly correlated with the MRI parameters (see Figure 3). However, applying cardiac and/or respiratory gating may partly compensate the lower image quality. Our experience (unpublished) with gating is that it seems to result in low signal-to-noise ratio images due to low count density. Administrating higher doses of radioactivity could potentially compensate this issue. However, two problems would arise: (1) higher doses require larger elution volumes precluding the use in small animals. (2) higher doses would also increase random and scatter effects, in addition to greater dead-time artifacts. The Inveon scanner user guide recommends no higher injected dose than 100 to 120 MBq in order to obtain the best noise equivalent count rate (NECR). However, further investigation into post-imaging reconstruction and processing is warranted.

It could be speculated that the reduced ^82^Rb-uptake in the infarcted regions is the result of the residual partial volume effect caused by the thinning of the myocardial wall after infarction. However, while the anterolateral wall of many rats had diminished wall motion as observed by MRI cine, no outright wall thinning was observed at the follow-up MRI scans.

MRI was selected as reference due to its high spatial resolution and high reproducibility in LV function and morphology estimations.^17^ Furthermore, MRI allows assessment of the infarct size with LGE, which has been validated against histology in rats.^15^ MRI-derived parameters are associated with morbidity and mortality in the clinical setting, which justifies MRI as Ref. 18 As expected, we found larger infarcts in the permanent ligation group compared to the ischemia-reperfusion group. Our results were very similar to the non-invasive and histological estimations of Luo et al for both intervention methods,^15^ which in combination with the high degree of inter-observer reproducibility, supports the validity of our MRI results. For both the permanent ligation and the ischemia-reperfusion group, the correlations for follow-up ^82^Rb-uptake were strongest with the MRI-derived infarct sizes as in contrast to the LVEF. This suggests that the ^82^Rb activity is a better surrogate marker of infarct size than LV function. In contrast to the permanent ligation group, the follow-up ^82^Rb-uptake of the ischemia-reperfusion group did not significantly correlate to follow-up LVEF. This was probably the result of the limited reduction of ^82^Rb activity at follow-up due to variation in the degree and extent of ischemia applied. Furthermore, the relative limited resolution and blurring impede the detection of small perfusion defects. Hence, underscoring the need for improved post-imaging processing to reducing blurring and blood-myocardium cross-contamination.

^13^N-ammonia perfusion tracer has previously been used successfully in rats and mice to depict perfusion defects.^9^,^19^ However, both studies only conducted imaging when the artery was totally occluded and not after ischemia-reperfusion. While ^13^N-ammonia produces images with better spatial resolution compared to ^82^Rb because of less scatter and shorter positron range, ^82^Rb-imaging has two main advantages: first, no need of onsite cyclotron, and second, a higher throughput due to a faster imaging protocol. However, the continuing cost of an ^82^Rb-generator must be kept in mind.

^82^Rb-imaging has become the standard perfusion tracer in many PET centers and has been validated against other perfusion tracers and histology.^20^–^22^ However, important inherent traits of ^82^Rb are unknown regarding tracer kinetics and extraction during hyperemia, and metabolic derangement after ischemia-reperfusion injury.^23^ Furthermore, the impact of many standard medication regiments in coronary artery disease (e.g., statins, antihypertensive, anticoagulants) on ^82^Rb-uptake is unknown. Nevertheless, our model seems promising and with further improvement in reconstruction processing could enable preclinical testing with various drugs and improve the pathophysiological understanding of the ^82^Rb-uptake in myocardial tissue free of injury and after infarction.

## Limitations

This study has several limitations. One being that preconditioning of the heart in the clinical setting plays an important role but was absent in our setup. Our model of ischemia-reperfusion is comparable to the clinical setting, but does not adequately address the preconditioning issue. However, consecutive opening and closing of the snare before total occlusion can simulate this effect. Second, the in vivo results were not compared to ex vivo measurements, which could validate the methodology further. However, the MRI, which was used as reference in this study, has previously been validated with histology.^15^

Despite the calibration of the ^82^Rb-generator before each use, the exact amount of tracer activity injected in each rat is difficult to estimate because of the manual handling of the dose infusion and rapid radioactivity decay. Moreover, the species-specific myocardial uptake of the ^82^Rb in rats is unknown and could be different from humans. However, ^82^Rb blood flow measurements investigated in canine models were comparable with microsphere blood flow measurements.^23^

## Conclusion

To the best of our knowledge, this study is the first to examine the feasibility of ^82^Rb-PET imaging and comparing it to MRI-derived measurements in rats. In a chronic and an ischemia-reperfusion rat model, the infarct size correlated significantly with the ^82^Rb-uptake. Furthermore, the segmental ^82^Rb-uptake after infarction predicted the wall motion and LGE enhancement as measured by MRI. Further studies are needed to define the accuracy, precision, and repeatability of ^82^Rb-perfusion imaging in rats. These results may with enhanced post-imaging processing encourage preclinical serial evaluation of the myocardial perfusion in a time-efficient manner.

## New Knowledge Gained

^82^Rb-PET can be utilized to produce myocardial perfusion images in rats, and enhance the cost–benefit profile of clinically available ^82^Rb-generator.

## Electronic supplementary material

Below is the link to the electronic supplementary material.

Supplementary material 1 (PPTX 3154 kb)

## Abbreviations

AHA-17: American Heart Association 17 segment model
CT: Computed tomography
EDV: End-diastolic volume
ESV: End-systolic volumes
LAD: Left anterior descending artery
LGE: Late gadolinium enhancement
LV: Left ventricle
LVEF: Left ventricle ejection fraction
MI: Myocardial infarction
MRI: Magnetic resonance
OSEM: Ordered-subsets expectation maximization
PET: Positron emission tomography
ROI: Region of interest
TR: Repetition time
TE: Echo time
SPECT: Single-photon emission computed tomography
^82^Rb: Rubidium-82
