# Supplementary material for: Rubidium-82 PET imaging is feasible in a rat myocardial infarction model
Source: J Nucl Cardiol. 2017 Jul 18;26(3):798–809. doi: 10.1007/s12350-017-0994-9 (PMC6517336; doi:10.1007/s12350-017-0994-9)
Supplement: Supplementary file 1 — Supplementary material 1 (PPTX 3154 kb) [file 12350_2017_994_MOESM1_ESM.pptx]

## Slide 1
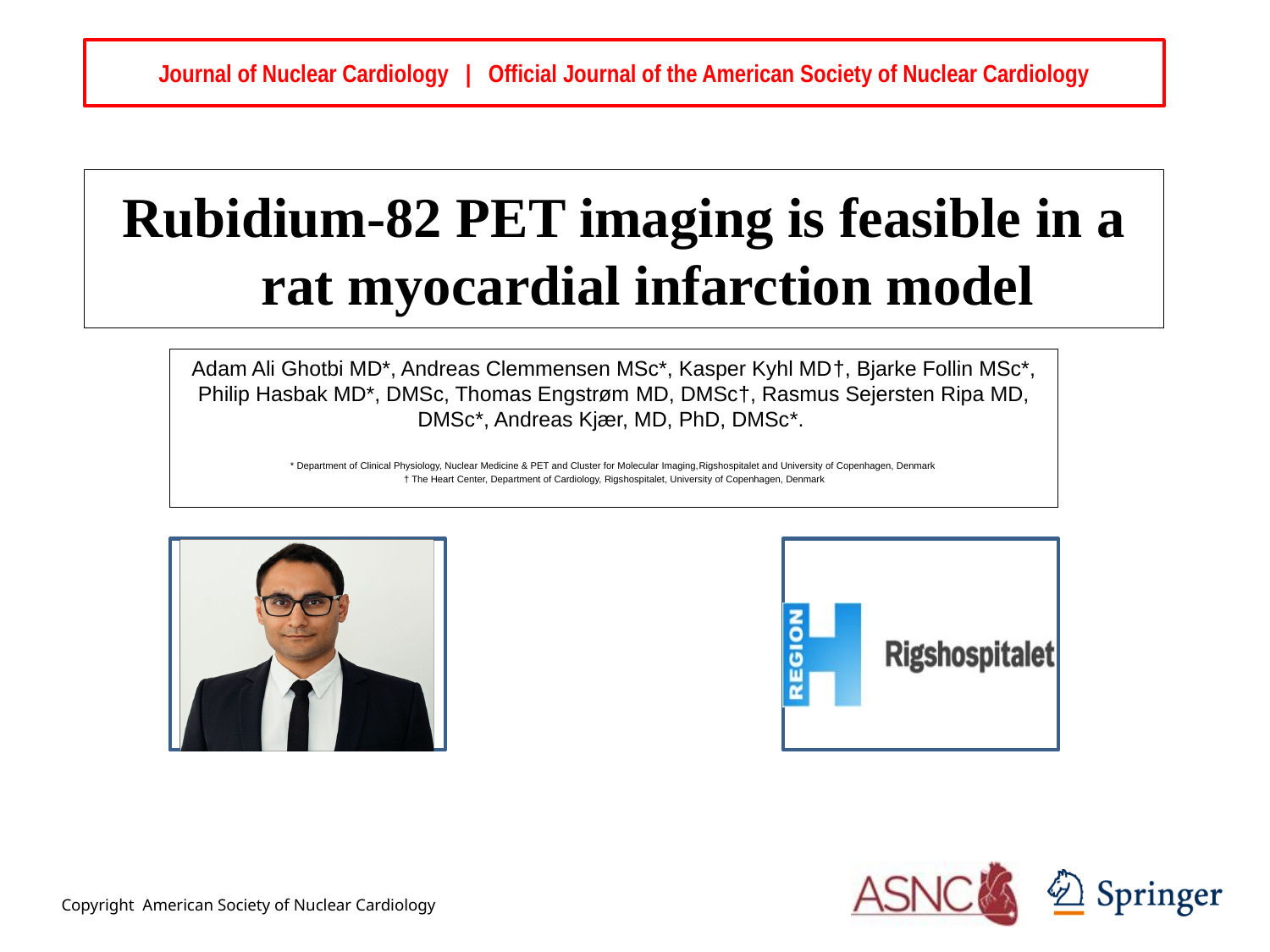

Journal of Nuclear Cardiology | Official Journal of the American Society of Nuclear Cardiology
# Rubidium-82 PET imaging is feasible in a rat myocardial infarction model
Adam Ali Ghotbi MD*, Andreas Clemmensen MSc*, Kasper Kyhl MD†, Bjarke Follin MSc*, Philip Hasbak MD*, DMSc, Thomas Engstrøm MD, DMSc†, Rasmus Sejersten Ripa MD, DMSc*, Andreas Kjær, MD, PhD, DMSc*.
* Department of Clinical Physiology, Nuclear Medicine & PET and Cluster for Molecular Imaging, Rigshospitalet and University of Copenhagen, Denmark
† The Heart Center, Department of Cardiology, Rigshospitalet, University of Copenhagen, Denmark
Head shot of author
required
Copyright American Society of Nuclear Cardiology

## Slide 2
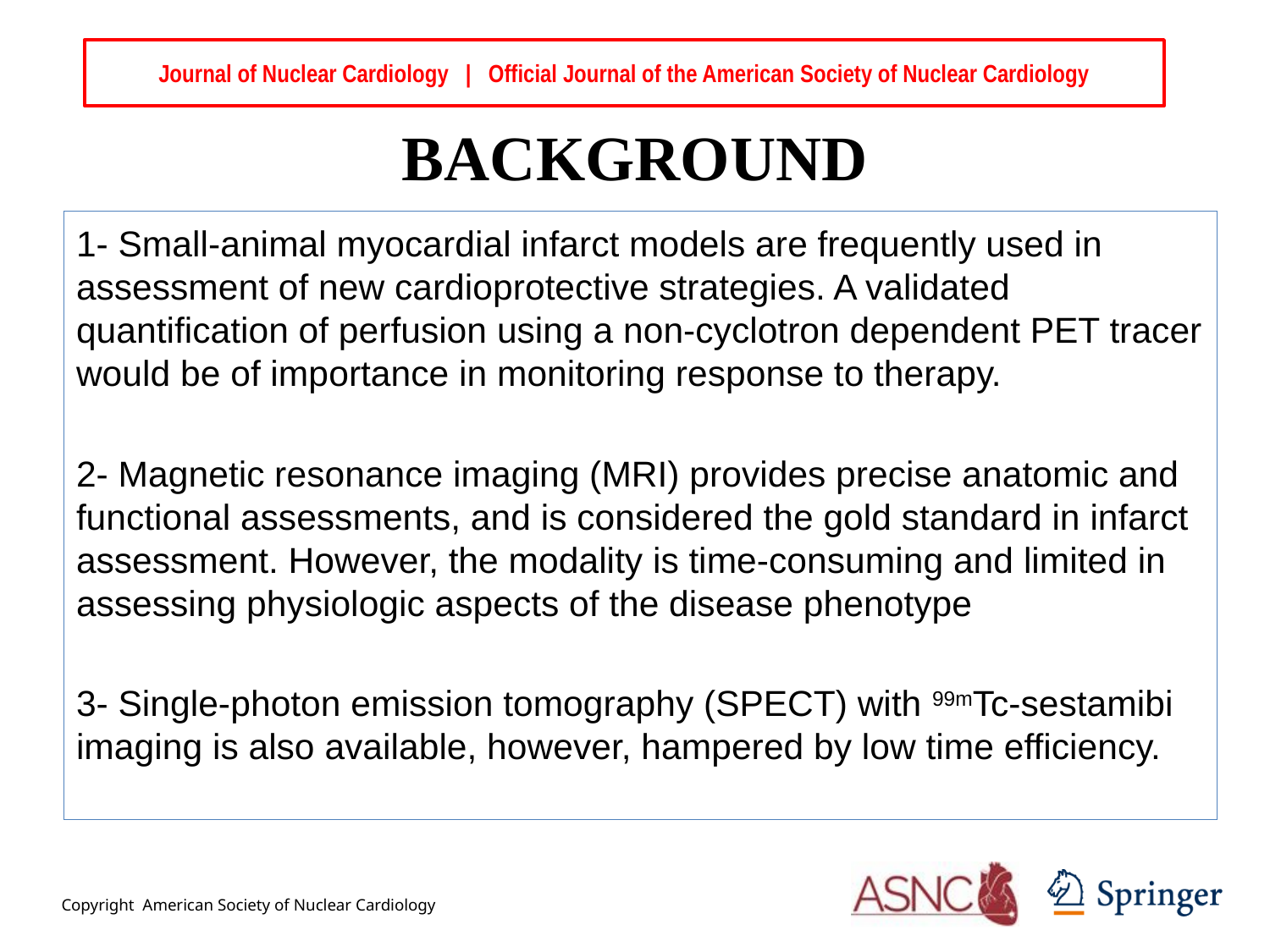

Journal of Nuclear Cardiology | Official Journal of the American Society of Nuclear Cardiology
# BACKGROUND
1- Small-animal myocardial infarct models are frequently used in assessment of new cardioprotective strategies. A validated quantification of perfusion using a non-cyclotron dependent PET tracer would be of importance in monitoring response to therapy.
2- Magnetic resonance imaging (MRI) provides precise anatomic and functional assessments, and is considered the gold standard in infarct assessment. However, the modality is time-consuming and limited in assessing physiologic aspects of the disease phenotype
3- Single-photon emission tomography (SPECT) with 99mTc-sestamibi imaging is also available, however, hampered by low time efficiency.
Copyright American Society of Nuclear Cardiology

## Slide 3
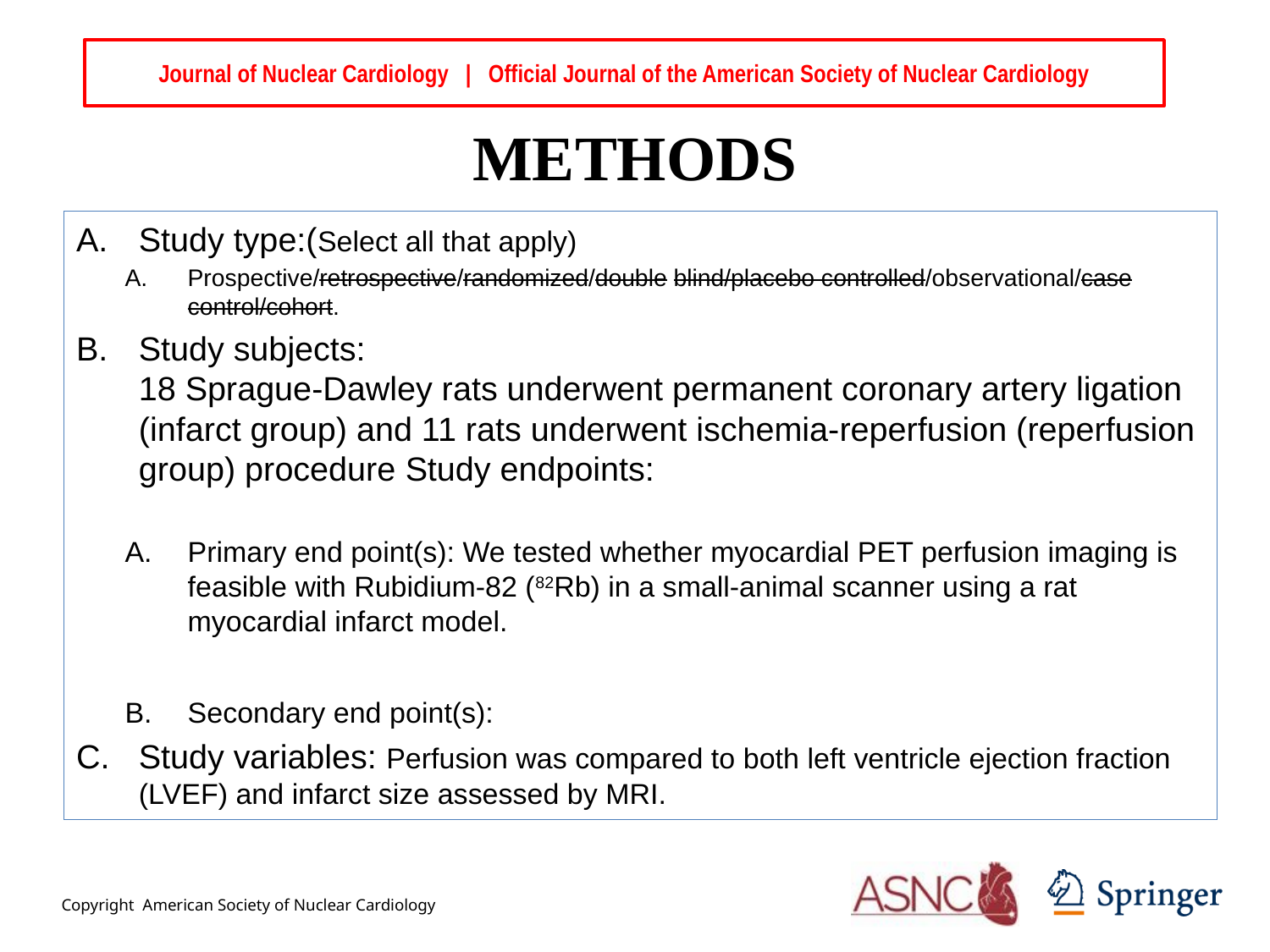

Journal of Nuclear Cardiology | Official Journal of the American Society of Nuclear Cardiology
# METHODS
Study type:(Select all that apply)
Prospective/retrospective/randomized/double blind/placebo controlled/observational/case control/cohort.
Study subjects:
18 Sprague-Dawley rats underwent permanent coronary artery ligation (infarct group) and 11 rats underwent ischemia-reperfusion (reperfusion group) procedure Study endpoints:
Primary end point(s): We tested whether myocardial PET perfusion imaging is feasible with Rubidium-82 (82Rb) in a small-animal scanner using a rat myocardial infarct model.
Secondary end point(s):
Study variables: Perfusion was compared to both left ventricle ejection fraction (LVEF) and infarct size assessed by MRI.
Copyright American Society of Nuclear Cardiology

## Slide 4
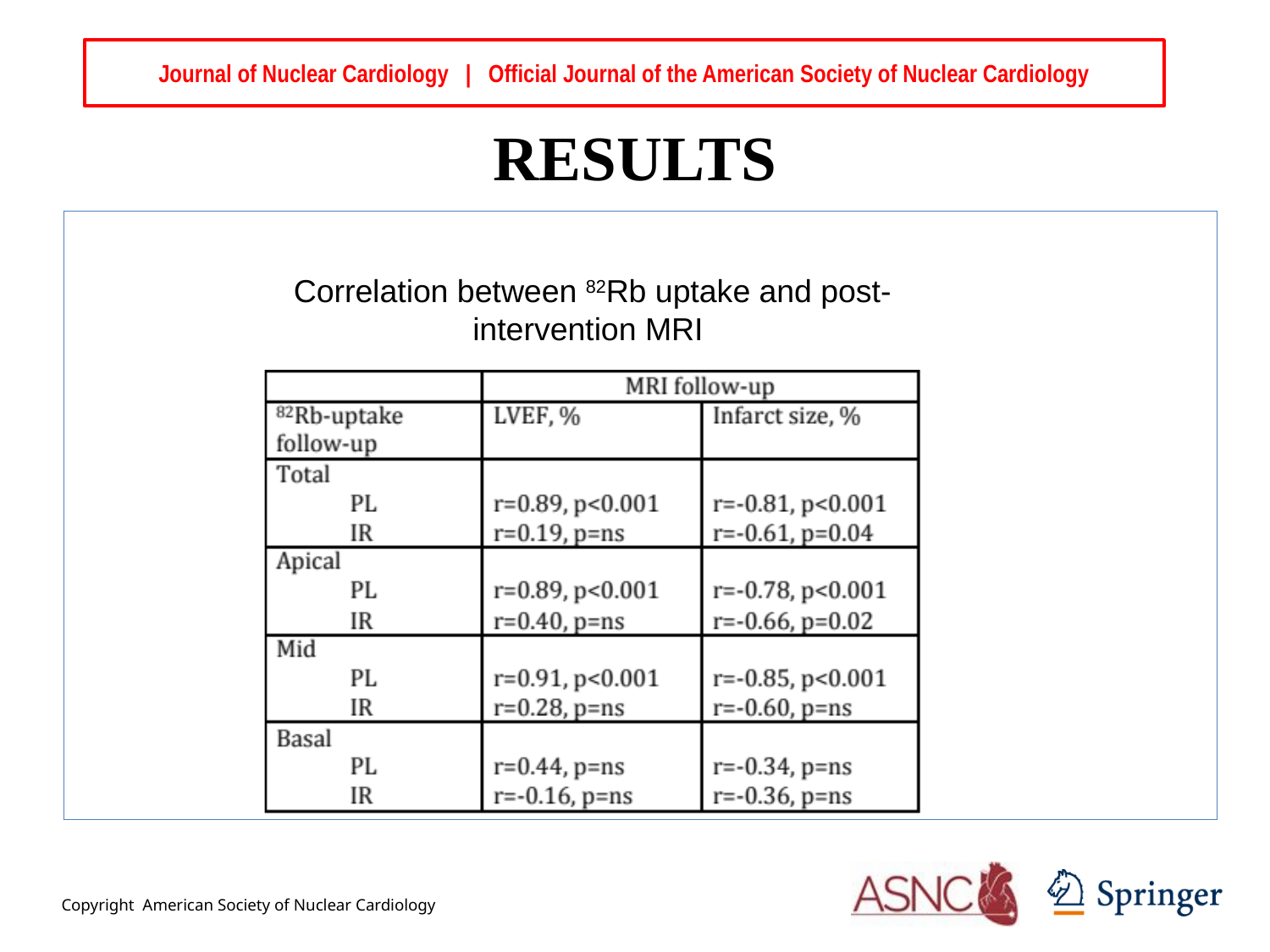

Journal of Nuclear Cardiology | Official Journal of the American Society of Nuclear Cardiology
# RESULTS
Correlation between 82Rb uptake and post-intervention MRI
Copyright American Society of Nuclear Cardiology

## Slide 5
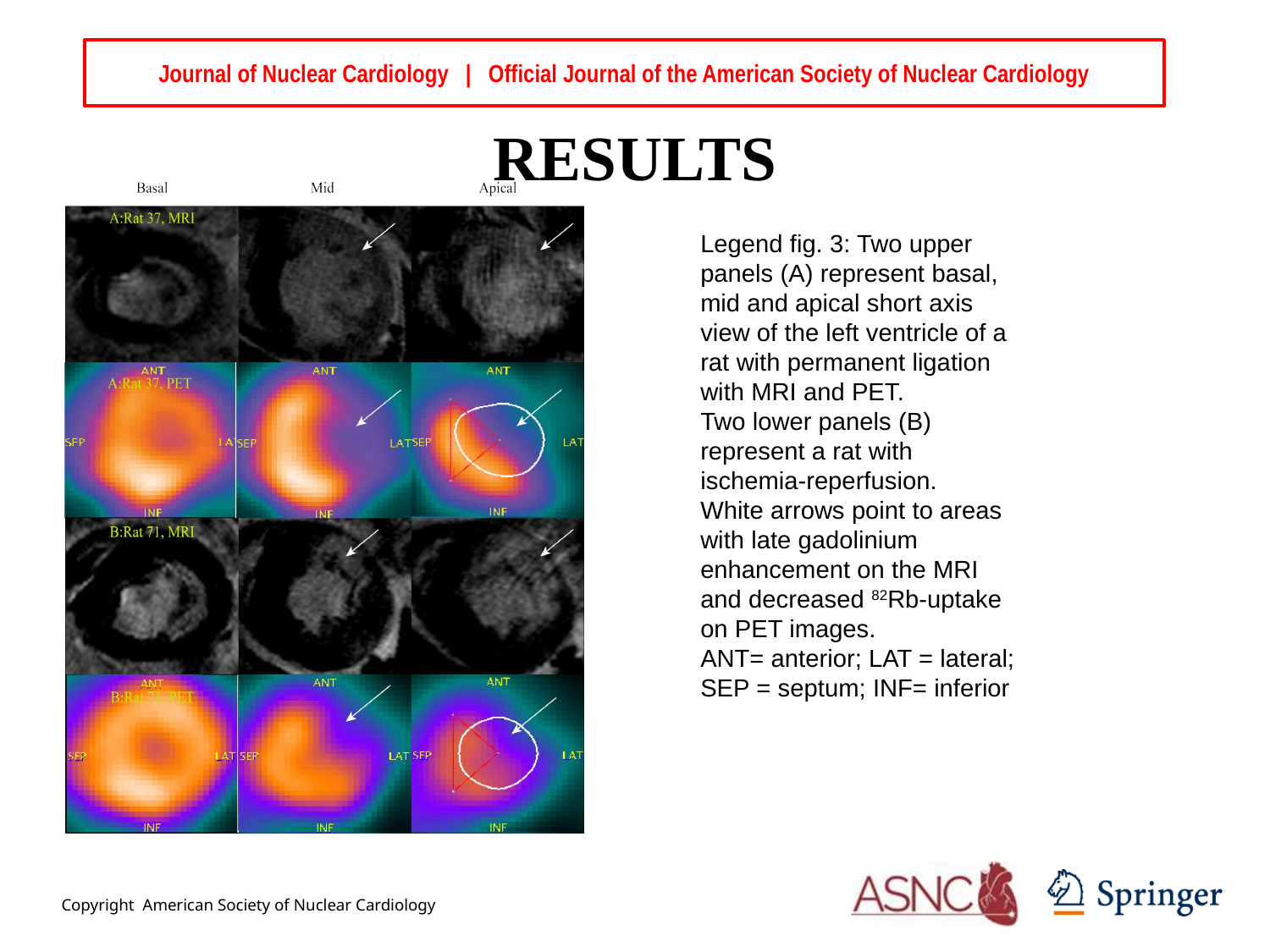

Journal of Nuclear Cardiology | Official Journal of the American Society of Nuclear Cardiology
# RESULTS
Legend fig. 3: Two upper panels (A) represent basal, mid and apical short axis view of the left ventricle of a rat with permanent ligation with MRI and PET.
Two lower panels (B) represent a rat with ischemia-reperfusion.
White arrows point to areas with late gadolinium enhancement on the MRI and decreased 82Rb-uptake on PET images.
ANT= anterior; LAT = lateral; SEP = septum; INF= inferior
Copyright American Society of Nuclear Cardiology

## Slide 6
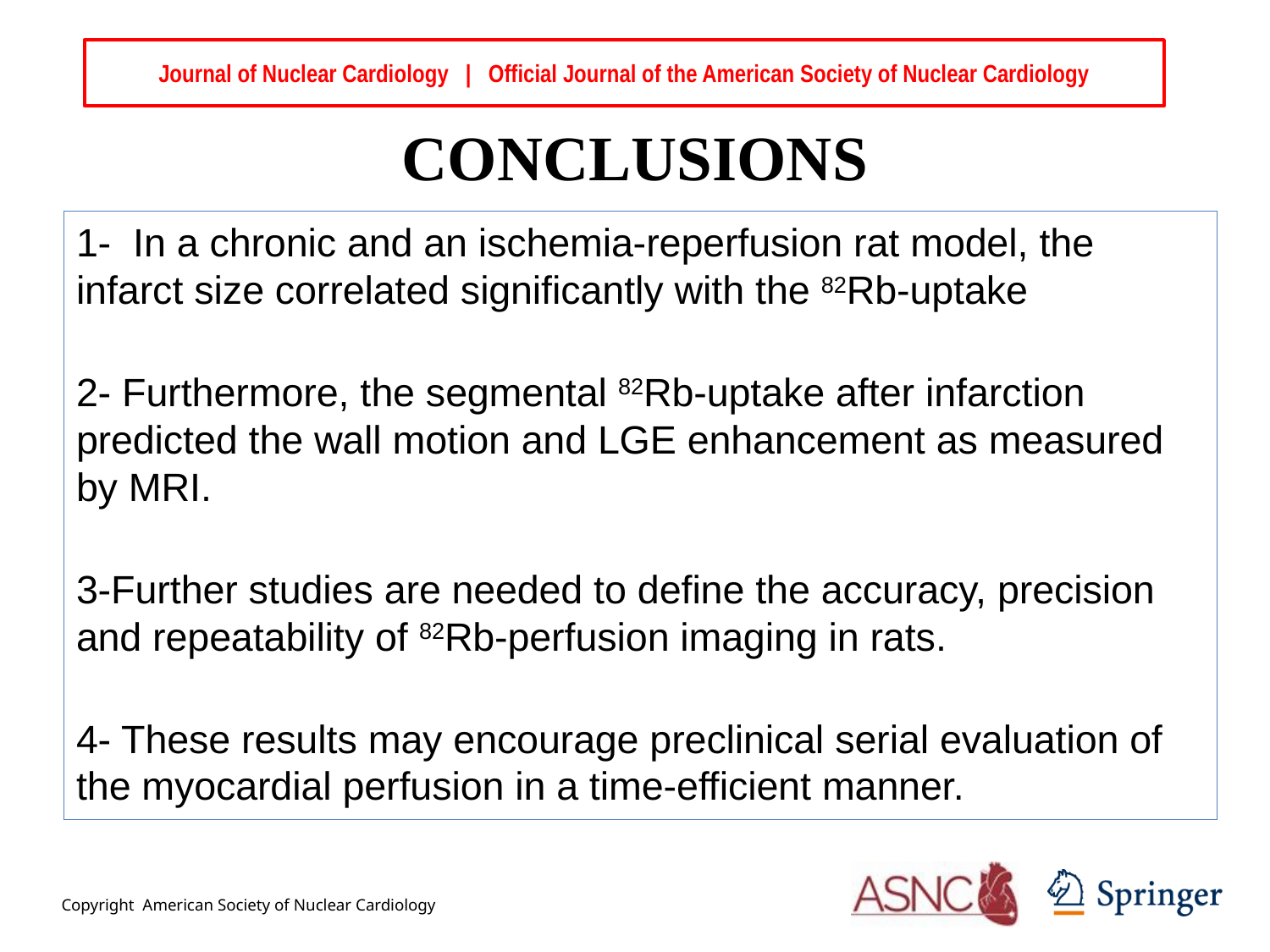

Journal of Nuclear Cardiology | Official Journal of the American Society of Nuclear Cardiology
# CONCLUSIONS
1- In a chronic and an ischemia-reperfusion rat model, the infarct size correlated significantly with the 82Rb-uptake
2- Furthermore, the segmental 82Rb-uptake after infarction predicted the wall motion and LGE enhancement as measured by MRI.
3-Further studies are needed to define the accuracy, precision and repeatability of 82Rb-perfusion imaging in rats.
4- These results may encourage preclinical serial evaluation of the myocardial perfusion in a time-efficient manner.
Copyright American Society of Nuclear Cardiology
